# Hydrostatic pressure regulates CYP1A2 expression in human hepatocytes via a mechanosensitive aryl hydrocarbon receptor-dependent pathway

**DOI:** 10.1152/ajpcell.00472.2019

**Published:** 2020-03-11

**Authors:** Lewis Burton, Paula Scaife, Stuart W. Paine, Howard R. Mellor, Lynn Abernethy, Peter Littlewood, Cyril Rauch

**Affiliations:** ^1^School of Veterinary Medicine and Science, University of Nottingham, Sutton Bonington, United Kingdom; ^2^Division of Medical Sciences and Graduate Entry Medicine, School of Medicine, University of Nottingham, Royal Derby Hospital Centre, Derby, United Kingdom; ^3^Vertex Pharmaceuticals Europe Ltd., Abingdon Oxfordshire, United Kingdom

**Keywords:** drug metabolism, hepatocytes, mechanosensitivity

## Abstract

Approximately 75% of xenobiotics are primarily eliminated through metabolism; thus the accurate scaling of metabolic clearance is vital to successful drug development. Yet, when data is scaled from in vitro to in vivo, hepatic metabolic clearance, the primary source of metabolism, is still commonly underpredicted. Over the past decades, with biophysics used as a key component to restore aspects of the in vivo environment, several new cell culture settings have been investigated to improve hepatocyte functionalities. Most of these studies have focused on shear stress, i.e., flow mediated by a pressure gradient. One potential conclusion of these studies is that hepatocytes are naturally “mechanosensitive,” i.e., they respond to a change in their biophysical environment. We demonstrate that hepatocytes also respond to an increase in hydrostatic pressure that, we suggest, is directly linked to the lobule geometry and vessel density. Furthermore, we demonstrate that hydrostatic pressure improves albumin production and increases cytochrome *P*-450 (CYP) 1A2 expression levels in an aryl hydrocarbon-dependent manner in human hepatocytes. Increased albumin production and CYP function are commonly attributed to the impacts of shear stress in microfluidic experiments. Therefore, our results highlight evidence of a novel link between hydrostatic pressure and CYP metabolism and demonstrate that the spectrum of hepatocyte mechanosensitivity might be larger than previously thought.

## INTRODUCTION

Currently, the intrinsic clearance of a drug is determined in vitro using hepatoma cell lines, isolated primary hepatocytes, or enriched subcellular fractions and scaled up to in vivo using mathematical models (7, 58). However, it is becoming increasingly common for hepatocyte assays to underpredict metabolic clearance even after empirical corrections (26), with inadequate scaling and predictions potentially leading to a poor transition between in vitro and in vivo environments. Indeed, with pharmacokinetic and toxicity issues accounting for ~45% of total failures (63), the drawbacks involved in the use of isolated primary or hepatic-derived cell lines in generic two-dimensional culture conditions have become increasingly apparent.

**Fig. 1.**
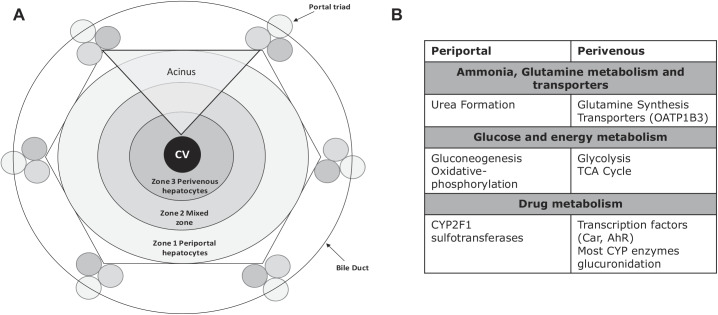
*A*: schematic representation of liver zonation from the periphery to the central vein (CV). *B*: increases in the gene expressed by hepatocytes located at the periphery or the center of the liver lobule (3, 6, 23, 25, 32, 33, 35). CYP2F1, cytochrome *P*-450 (CYP) 2F1; OATP1B3, organic anion-transporting polypeptide 1B3; TCA, tricarboxylic acid; CAR, constitutive androstane receptor; AhR, aryl hydrocarbon receptor.

In the body, hepatocytes subspecialize based on their pericentral positioning within the liver lobule (Fig. 1*A*), a functional unit within the liver. Hepatocytes closer to the central vein carry out a higher proportion of drug metabolism (24), while those closer to the periphery carry out more ammonia detoxification (Fig. 1*B*) (3). Thus, determination of the in vivo signals that contribute to hepatocyte specialization could prove paramount to elucidation of the underpredictions commonly observed when data are scaled from an in vitro environment and to improvement of pharmacokinetic modeling. When hepatocytes are isolated, they begin to dedifferentiate and despecialize; both of these events result in lower levels of drug-metabolizing enzymes (24, 29).

The loss of functionality, specification, and morphology that occurs when hepatocytes are cultured in vitro is well documented (2, 56) and has been attributed to the culture conditions in which they are grown. Attempts to maintain in vivo phenotype and functionality by restoring aspects of the in vivo physiological environment have met with mixed success. These attempts include controlling CO_2_ and O_2_ levels (31), culturing in three-dimensional tissue culture plates (55), supplementing growth medium with growth factors and cytokines (13, 54, 56), and coculturing with supportive cell types (59). More recent studies have found that culturing cells under flow, with the aim of reproducing the biophysical environment of hepatocytes, improves the phenotype and expression of metabolic enzymes and transporters (11, 60).

Various methods of flow application used on human, mouse, and rat hepatocytes and HepG2 cells have demonstrated increased cytochrome *P*-450 (CYP) 1A1/2 (CYP1A1/2) expression/activity and albumin production as one of their major findings (11, 17, 19, 41, 53, 60), with one study even alluding to the involvement of aryl hydrocarbon receptor (AhR) activation in the improvement of CYP1A1 in HepG2 cells via shear stress (41).

All published studies have concurred that altering the biophysical properties of cell culture, e.g., using basic hydrodynamics concepts, is somehow paramount to a better understanding of pharmacokinetics. However, how hydrodynamic stress increases the expression and/or activity of CYP enzymes at the lobule level is not well understood. Basic hydrodynamics, i.e., Poiseuille’s law, involves at least two fundamental physical parameters that balance each other, the pressure gradient and the shear stress, both of which are oriented along the flow. However, the assumption that the stresses are oriented only along the flow is legitimate as long as the vessels or capillaries have solid walls, i.e., the endothelial structure is solid. On the contrary, living tissues are soft and deformable under pressure, and, in this context, the notion of “wall stress,” otherwise known as Laplace’s law, becomes central. The wall stress results from a pressure applied against the vessel wall, i.e., with a component perpendicular to the flow. Therefore, the pressure gradient inside vessels generates a (hydrostatic) pressure with a component perpendicular to the flow at every position along vessels. As vessels are embedded in tissues, this suggests that irrigated tissues are under pressure locally at a magnitude that is a function of vessel density. This point has been suggested when the lobule vessels are modeled as a porous system (14), and it is in this context that we aimed to clarify the role of the hydrostatic pressure in hepatocyte function. Studies have demonstrated that the capillary hydrostatic pressure magnitude is ~1–2 kPa (37) and that both hydrostatic pressure and shear force are capable of modulating the lumen of circulatory vessels (12). Indeed, experimental work in rat and mouse liver has shown that the size of the sinusoidal vessels increases toward the central vein (10, 32, 46, 64), suggesting that pericentral hepatocytes experience compression.

Here, we show that hydrostatic pressure is sufficient to increase albumin production and the expression and activity of CYP1A2 relative to the nonpressurized control. Additionally, we demonstrate that the coincubation of pressurized hepatocytes with CH223191, an AhR antagonist (8, 66), is sufficient to inhibit the pressure-mediated increase in CYP1A2, suggesting that AhR could be a mechanosensitive nuclear receptor, which was later confirmed with a HepG2 reporter cell line. The effects of biophysical forces on hepatocytes are not completely novel; however, to the best of our knowledge, no work that links hydrostatic pressure to improved hepatocyte functionality has been conducted.

## MATERIALS AND METHODS

### 

#### Ethical considerations.

The pig liver tissues were obtained from animals euthanized for a nonresearch purpose.

#### Collection and staining of liver tissue.

A ~5 × 5 cm^2^ section from three separate freshly slaughtered pigs was collected from Elliot and Son’s abattoir, immediately placed in formalin buffer, and left overnight. The tissue was then processed and stained using traditional methods (21). The vessels, hepatocytes, nuclei, and branch points were separated based on threshold parameters (see Supplemental Material, available at https://doi.org/10.6084/m9.figshare.11923188) in Image-Pro Plus software. The area and centroid coordinates of the objects were extracted and used to calculate the physical parameters (see detailed instructions for separation and calculation in Supplemental Material). Images were taken at ×10 magnification with a DM5000 B microscope (Leica) using a DFC420 camera (Leica) and Leica Application Suite (LAS) software (version 3.8).

#### Thawing and seeding cryopreserved human hepatocytes and HepaRG cells.

LiverPool cryoplateable human hepatocytes (10-donor pool) were purchased from BioVit. Hepatocytes were thawed in a water bath preheated to 37°C for 1–2 min and placed into a class II tissue culture hood. The cell suspension was carefully transferred to a 50-mL centrifuge tube containing preheated cryopreserved hepatocyte recovery medium (Thermo Fisher), which was centrifuged at 100 *g* for 10 min at 20°C, and the supernatant was discarded. The cell pellet was resuspended in 5 mL of cryopreserved hepatocyte plating medium (Thermo Fisher). A 50:50 mix of cell suspension and Trypan blue solution was pipetted into a Countess cell-counting slide, and cell viability and concentration were determined automatically via the Countess automated cell counter. Hepatocytes were made up to 1 × 10^6^ cells/mL, and 300 µL were seeded into the wells of the pressure plate (see below). The hepatocytes had been allowed to adhere (~4 h), and the plating medium was exchanged for William’s E medium (Invitrogen) supplemented with hepatocyte maintenance cocktail (Thermo Fisher).

#### Setting up the pressure plate.

**Fig. 2.**
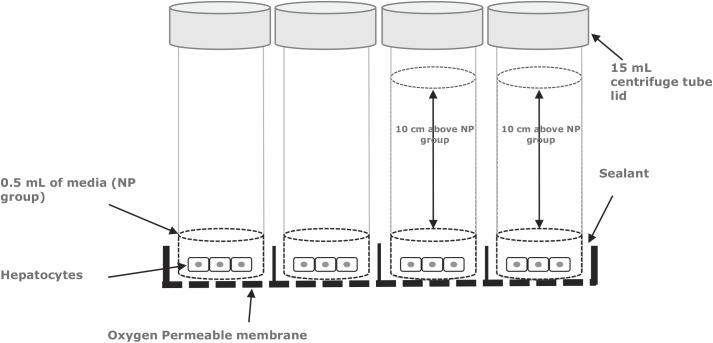
Schematic of the custom-made pressure plate. NP, no pressure.

Bottoms were removed from polystyrene tubes (15.5 mm outer diameter; product no. 55.461, Sarstedt), and the tubes were fitted into the wells of a 24-well Lumox plate, which has a gas-permeable membrane and allowed gases to diffuse through the base of the plate directly to the cell monolayer. The tubes were glued and sealed to the plate using laboratory-grade silicone sealant (Dow Corning) and left to cure for 72 h at 37°C. The resulting construct was sterilized in a UV cross-linker (catalog no. CL-1000, UVP) at 5,000 µJ/cm^2^ for 60 min. The wells were then coated with collagen I solution from rat tail (5 µg/cm^2^; Sigma Aldrich) and allowed to dry. The wells were washed five times with 5 mL of sterile Dulbecco’s PBS (DPBS) buffer, and hepatocytes were seeded at 3 × 10^5^ cells/well and left to adhere overnight. On the following morning, the medium was removed, and the cells were exposed to either 0.28 cm (500 µL) of William’s E medium [no-pressure (NP) group] or 10 cm (12.5 mL) of William’s E medium [with-pressure (WP) group] for a maximum of 72 h, as shown in Fig. 2.

#### Cell imaging.

For imaging, the medium was aspirated, and the sealant (Dow Corning) was removed using a sterile scalpel blade, isopropanol, and paper wipes. The tubes were carefully removed from the plate, and the cell monolayer was washed three times with DPBS and imaged on an EVOS FL cell-imaging system (Thermo Fisher Scientific); two images were taken per well. The phenotype of the hepatocytes was examined nonquantitatively.

#### Water-soluble tetrazolium salt assay.

Water-soluble tetrazolium salt (WST-1) solution, a mixture of 10% (vol/vol) WST-1 (Roche) and cell culture medium, was applied to the cell monolayer. After 1 h of incubation, 80 µL of solution were spiked into a 96-well plate, and optical density at 440 nm was read on a PHERAstar FSX plate reader.

#### Albumin assay.

After incubation, two 300-µL samples of medium from each condition were frozen at −80°C for later analysis. Albumin was detected using a human albumin sandwich ELISA kit (Abcam) following the manufacturer’s instructions. Each condition was read in duplicate for each sample. The absorbance was read on a PHERAstar FSX plate reader (BMG Labtech).

#### Lactate dehydrogenase release assay.

Lactate dehydrogenase (LDH) release was assessed calorimetrically using the Pierce LDH cytotoxicity assay kit (Thermo Fisher Scientific) following the manufacturer’s instructions. Duplicate samples were taken from each well and averaged, and the absorbance was read on the PHERAstar FSX plate reader (BMG Labtech). Data are shown as percent viability, with cells treated with 1× LDH lysis buffer as 0% viable and an empty well containing collagen and medium as 100% viable. Percent viability was calculated using the following equation

$$\text{Viability }\left(\%\right)=100-\frac{\left(\text{sample}-100\text{\% control}\right)}{0\text{\% Control}}\times 100.$$

#### Pierce bicinchoninic acid protein recovery assay.

After incubation, the medium was removed, cells were washed three times with DPBS, and the monolayer was lysed with radioimmunoprecipitation assay buffer (Thermo Fisher). The protein concentration was calculated using a Pierce bicinchoninic acid (BCA) protein assay kit (Thermo Fisher) following the manufacturer’s instructions. Percent protein recovery was calculated assuming that *T*_0_ is equal to 100% as follows

$$\text{Protein recovery }\left(\text{\%}\right)=\;\frac{\text{Protein Concentration }T_{x}}{\text{Protein Concentration }T_{0}}\times \text{1}00$$

#### RNA extraction and cDNA conversion.

After incubation, the monolayer was washed, and RNA was extracted using the SV96 total RNA isolation system (Promega) via a vacuum-based method following the manufacturer’s instructions. Once extracted, RNA was stored at −80°C until used. Quantification of RNA concentration was done using the Quant-IT RNA assay kit following the manufacturer’s instructions (Invitrogen). cDNA conversion and quantitative PCR (qPCR) were carried out using the GoTaq two-step RT-qPCR kit (Promega) with a final concentration of 0.5 ng/µL.

#### Quantitative PCR.

GoTaq 2× master mix was mixed with nuclease-free water, the gene of interest [CYP3A4, CYP1A2, CYP2B6, CYP2C9, CYP2D6, aldehyde oxidase 1 (AOX1), hypoxanthine-guanine phosphoribosyltransferase 1 (HPRT1), or GAPDH (TaqMan Gene Expression Assay, Thermo Fisher Scientific], and cDNA template at a ratio of 10:7:1:2 in an RNase/DNase-free tube. The final volume of 20 µL containing 1 ng of cDNA stock was added to each well of the qPCR multiwell plate (Agilent). Each condition was triplicated: the plate included triplicates of no-reverse transcriptase (NRT) and no-template controls, which contained 2 µL of nuclease-free water in the place of the cDNA template. The probe-based dye setting and the quick cycle parameters were used for the AriaMx real-time PCR system (Agilent). Data were collected, exported, and processed using the 2^−ΔΔCT^ method (49). HPRT and GAPDH stability was assessed by visual comparison of cycle thresholds (C_T_) and standard deviations; the more stable (in all cases, HPRT) was used as the housekeeping gene.

#### P450-Glo metabolism assay.

Pressure was removed after 48 h, and the cell monolayer was washed three times with DPBS. The cells were then dosed with the appropriate luciferin conjugate substrate for 45 min. The solution was mixed 1:1 with luciferin detection reagent and read using the luminescence detection settings on the PHERAstar FSX plate reader (BMG Labtech). The cell monolayer was washed and lysed, and a protein assay was conducted as described above. The luminescence value was divided by total protein and displayed as increase in CYP activity relative to the NP group. Several pressure points were tested for their effects on metabolic activity (see Supplemental Fig. S1, available at https://doi.org/10.6084/m9.figshare.11923194)

#### AhR induction assay.

Pressure was applied as previously described, but medium was supplemented with 0.2% DMSO (vehicle), 50 µM omeprazole (Sigma Aldrich), or 10 µM CH223191 (Sigma Aldrich) and exchanged every 24 h. After the 48-h incubation period, pressure was removed, and CYP1A2 expression and activity were assayed by P450-Glo and qPCR, as previously described.

#### DRE-1A2 AhR reporter cell line.

The DRE-1A2 HepG2-derived reporter cell line was purchased from Puracyp, and luciferin-1A2 was purchased from Promega. A CYP1A2 activity and AhR activation assays were conducted following the manufacturer’s protocol.

#### Statistics.

Data were assessed for normality using the Anderson-Darling test and visually by a *Q-Q* plot. Variance was assessed using Levene’s test, and data that did not appear normal were log-transformed and reassessed.

Linear regression of histology data was checked for a significant difference (*P* < 0.05) from an intercept-only model (i.e., the *x* parameter had no impact) using the *F* test. *P* < 0.05 was considered statistically significant.

Statistics on cell-based assays were conducted by a two-way ANOVA followed by Bonferroni’s post hoc test or, when comparing only two groups, Student’s unpaired *t* test. *P* < 0.05 was considered statistically significant.

## RESULTS

### 

#### Determination of biophysical parameters of the liver lobule relative to its pericentral positioning.

**Fig. 3.**
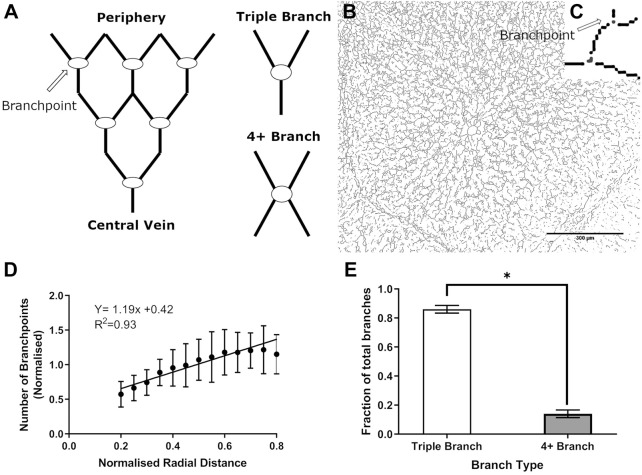
Number and type of branch points moving along the pericentral axis of the liver lobule. *A*: schematic of the convergence of vessels from the periphery toward the central vein (*left*) and branch types (*right*). *B* and *C*: skeletonized lobule (*B*) and higher-magnification image of a branch point exemplifying a typical triple branch detected by Image-Pro Plus (*C*). Scale bar = 300 µm. *D*: number of branch points as a function of radial distance. *E*: relative proportions of branch points in the liver lobule. Values are means (SD). Three animals were assessed (*n* = 3), with 9 lobules measured per animal, spread across 3 separate tissue sections for a total of 27 lobules analyzed. Significance was determined by unpaired *t* test (*P* < 0.05). Data in *D* were fitted to a linear regression equation and show significant improvement over an intercept-only model (*F* test).

**Fig. 4.**
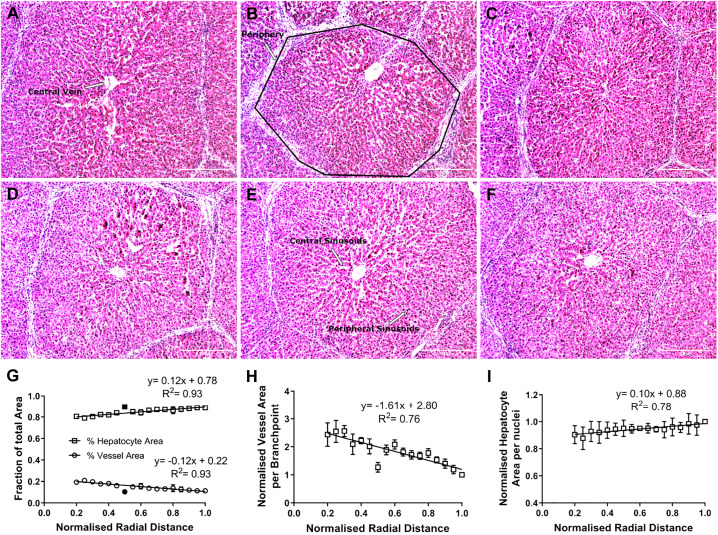
*A–F*: hematoxylin-eosin-stained sections, with regions of importance annotated. Data were fitted using GraphPad automatic outlier detection; outliers are displayed in black fill. Scale bars = 300 µm. *G*: fraction of total area occupied by hepatocytes (□) and vessels (○) as a function of their normalized distance (*R*) from the central vein. *H*: area of vessels per branch point decreased as a function of radial distance. *I*: area of hepatocytes per nucleus increased. Values are means (SD). Three animals were assessed (*n* = 3), with 9 lobules measured per animal, spread across 3 separate tissue sections for a total of 27 lobules analyzed. Data in *G–I* were fitted to a linear regression equation and show significant improvement over an intercept-only model (*F* test).

Pig liver tissue was used to assess the structure and physical parameters of the liver lobule. Pig tissue was chosen due to easy access to healthy liver tissue, which is important because disease states such as cirrhosis have been shown to increase the stiffness of liver tissue from between 0.3 and 0.6 kPa to a maximum of 12 kPa (23), which could alter physical properties. Additional consideration included tissue cost and closer similarity of pig than either rat or mouse liver to human liver (1). Use of thresholding methods (see Supplemental Materials and Methods) and image analysis software enabled segregation of the liver lobule into branch points (Fig. 3), hepatocytes, nuclei, and vessels (Fig. 4). The nuclei and branch points were used as an indirect measure of the number of hepatocytes and vessels, respectively. Data were plotted relative to proximity to the central vein (CV), where *R* = 0 is the center of the CV. The high collagen content of the pig lobule allows for easy visualization and separation of a lobule (Fig. 4*B*). Within a liver lobule, the center of the CV was set as the origin. The abundance of blood vessels, hepatocytes, nuclei, and branch points was quantified as a function of the distance from the CV.

More precisely, skeletonizing and thinning the lobule allowed the identification of vessel branch points, which were used as indirect measurements of vessel number, allowing quantification of the change in vessel number over radial distance. Figure 3*A* shows a schematic representation of the convergence of vessels from the periphery toward the CV and an example of three vs. four or more branch points identified by Image-Pro Plus software. The numbers of branch points (Fig. 3*D*) were fitted to a linear regression curve. The points after *R* = 0.8 were excluded in the linear regression calculation, because the diameters of lobule peripheries were heterogeneous among tissue sections analyzed, skewing the results toward the periphery. The *R*^2^ of the curve was 0.93, and an *F* test determined that this model was significantly better than an intercept-only model (*P* < 0.001). Results showed that distance from the CV significantly increased the number of branch points. An unpaired *t* test conducted on the proportion of branch types (Fig. 3*E*) revealed significantly more triple-branched points (*P* < 0.001), supporting the convergence model.

The data also show a decrease in the density of vessels and an increase in hepatocyte density upon moving from the CV (*R* = 0) to the periphery (*R* = 1) of the lobule (Fig. 4*G*). To verify this, the total area within the radial segments was divided by the sum of either branch points (for vessels) or nuclei (for hepatocytes) within those same segments (Fig. 4, *H* and *I*). This shows that vessels drastically decrease (Fig. 4*H*), whereas hepatocytes slightly increase, in size from the CV toward the periphery (Fig. 4*I*). Data were fitted to linear regression equations, and the models were tested against an intercept-only model using the *F* test, with *P* < 0.05 considered statistically significant. A significant (*P* < 0.001 in all cases) improvement over an intercept-only model is observed for Fig. 4, *G–I*.

#### Model of hepatocytes under pressure mediated by the liver blood flow.

**Fig. 5.**
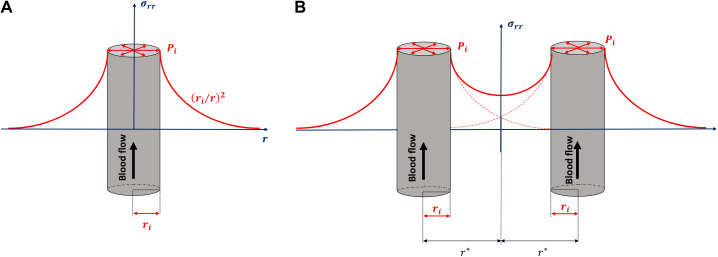
*A*: schematic of radial pressure, ${\text{σ}}_{\text{rr}}$, as a function of distance from the vessel. For simplicity of representation, the minus sign of the radial pressure has been omitted. *B*: if we now assume that 2 vessels are brought close to each other, then the radial pressure between vessels changes and the pressure applied on hepatocytes between vessels increases. Red dashed lines represent what would be the pressure for a single vessel. With use of notations developed in the text, the pressure between vessels will be a function of the distance separating vessels: ${\left(r_{i}/r^{*}\right)}^{2}$. However, ${\left(r_{i}/r^{*}\right)}^{2}$ can be rewritten as $\text{π}{\left(r_{i}\right)}^{2}/\text{π}{\left(r^{*}\right)}^{2}$. Given that $\text{π}{\left(r_{i}\right)}^{2}$ is the cross-sectional area of the vessel and that $\text{π}{\left(r^{*}\right)}^{2}$ is the surface area occupied by the vessel in the tissue, it follows that ${\left(r_{i}/r^{*}\right)}^{2}$ is the surface density of the vessel.

As stated in the introduction, the physical changes in the surface density of vessels, hepatocytes, and branch points may suggest that the lobule experiences pressure due to its anatomical ultrastructure. To model this, let us consider a single isolated vessel in a tissue with blood flowing within it, which is associated with a pressure gradient. The pressure inside the vessel is also applied against the vessel wall. Let us further concentrate on a small longitudinal section of the cylindrical vessel, such that the pressure inside this section is constant. From a physics point of view, the vessel is like a hollow cylinder in which an internal pressure can be defined. We shall assume that when the vessel is on its own, the pressure far away from the vessel is null. In this context, it is possible to demonstrate that the radial stress or radial pressure, ${\text{σ}}_{\text{rr}}$ (Fig. 5), on the tissue surrounding the vessel can be written as ${\text{σ}}_{\text{rr}}\sim -P_{i}\times {\left(r_{i}/r\right)}^{2}$, where $P_{i}$ is the internal pressure of the section of the vessel considered, $r_{i}$ is the radius of the vessel, and r is the distance from the center of the vessel (6). The minus sign in the expression of ${\text{σ}}_{\text{rr}}$ indicates a radial compression with a magnitude that decreases with the radial distance. Let us assume now that two vessels are next to each other; then the stress on the hepatocytes between the two vessels will increase as the distance between the vessels decreases (Fig. 5). Let us now focus on the variable ${\left(r_{i}/r\right)}^{2}$ and consider that the variable r is fixed to a typical value corresponding to the distance halfway between vessels. In this context, one shall define by $r^{*}$ this typical value. The variable defined by ${\left(r_{i}/r^{*}\right)}^{2}$ is then the surface density of vessels in the tissue or, similarly, the concentration of vessels in two dimensions. Therefore, provided that the pressure inside the vessels is constant, the higher the density of vessels, the higher the stress applied to hepatocytes between vessels. Of course, vessels are not necessarily parallel to each other, as shown schematically in Fig. 5, but this does not rule out the key conclusion that the density of vessels, whatever their orientations, defines the pressure applied to hepatocytes between vessels.

Naturally, when considering the blood flow in vessels in the liver, the pressure $P_{i}$ will vary along the vessels, being higher at the periphery and smaller at the center of the lobule. In addition, if the density of vessels changes from the periphery of the lobule to the CV, then, by noting R, the radial distance from the CV, the radial stress becomes a function of this new parameter and can be rewritten as ${\text{σ}}_{\text{rr}}\left(R\right)\sim -P_{i}\left(R\right)\times \text{α}\left(R\right)$, where $\text{α}\left(R\right)$ represents the density of vessels at the radial position R. Although the detailed and accurate knowledge of the three-dimensional ultrastructure of vessels in a lobule down to a scale of a few micrometers, similar to the size of hepatocytes, is still missing and, as a result, it is not possible to estimate with precision the variable defined by $P_{i}\left(R\right)$, two important results suggest that hepatocytes experience a compression toward the CV. The first result concerns the variable $\text{α}\left(R\right)$, which can be estimated experimentally by the percentage of vessel area as given in Fig. 4, *G* and *H*, and increases toward the CV. The second result is that if the hepatocytes experience a compression, it is expected that their surface area would be smaller. This latter point is shown experimentally in Fig. 4, *G* and *I*, demonstrating that the surface area of hepatocytes is smaller close to the CV.

Despite these limitations, it is still possible to estimate a ratio for the radial stresses. Indeed, for hepatocytes to experience a gradient of compression, the product $P_{i}\left(R\right)\times \text{α}\left(R\right)$ must change as a function of the radial position. Using a ratio for the radial stresses at two different radial locations, i.e., $R_{\text{min}}$ and $R_{\text{max}}$, in the lobule, one finds: $\frac{{\text{σ}}_{\text{rr}}\left(R_{\text{min}}\right)}{{\text{σ}}_{\text{rr}}\left(R_{\text{max}}\right)}\sim \frac{P_{i}\left(R_{\text{min}}\right)\times \text{α}\left(R_{\text{min}}\right)}{P_{i}\left(R_{\text{max}}\right)\times \text{α}\left(R_{\text{max}}\right)}$. Let us consider $R_{\text{max}}$ as the radial distance of the lobule periphery and $R_{\text{min}}$ as the radial distance close to the CV. Using Fig. 4*G*, one deduces $\frac{\text{α}\left(R_{\text{min}}\right)}{\text{α}\left(R_{\text{max}}\right)}\sim 1.9$. A value for $\frac{P_{i}\left(R_{\text{min}}\right)}{P_{i}\left(R_{\text{max}}\right)}\sim 0.7$ has been estimated using computational fluid dynamic modeling (43), meaning, in turn, that $\frac{{\text{σ}}_{\text{rr}}\left(R_{\text{min}}\right)}{{\text{σ}}_{\text{rr}}\left(R_{\text{max}}\right)}\sim 1.3$ or, equivalently, that the radial compression close to the CV increases by ~30%. It is in this context that pressure was imposed on isolated hepatocytes.

#### Effects of pressure on hepatocyte morphology.

**Fig. 6.**
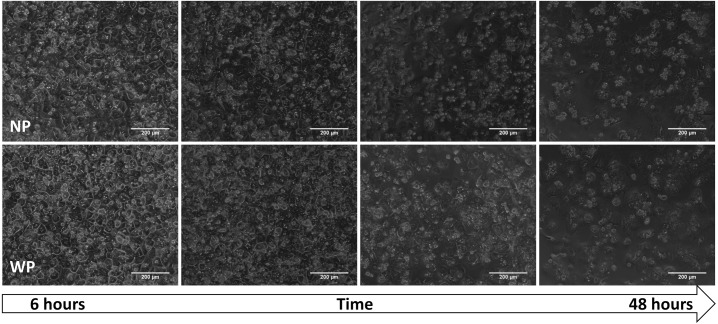
Bright-field images of hepatocytes after different incubation times with pressure (WP) or without pressure [no pressure (NP)]. Scale bars = 200 µm.

Hepatocytes were pressurized in a custom-made pressure plate (Fig. 2) after an incubation period under pressure or standard culture conditions. The pressure was removed, and the hepatocytes were imaged on an inverted microscope (Fig. 6). Differences in the phenotype of hepatocytes were assessed nonquantitatively. Attention was paid to visual markers of healthy differentiated hepatocytes: a bright nucleus, clear cell-cell junctions, and no gaps in the monolayer (44, 54). Hepatocytes in both conditions retained bright nuclei and a relatively confluent monolayer for the duration of culture (Fig. 6). On the contrary, cell-cell borders were most distinct at 6 h and became more difficult to observe over time. Overall, little difference between the NP and WP conditions was observed.

#### Effects of pressure on viability, albumin production, and protein recovery.

**Fig. 7.**
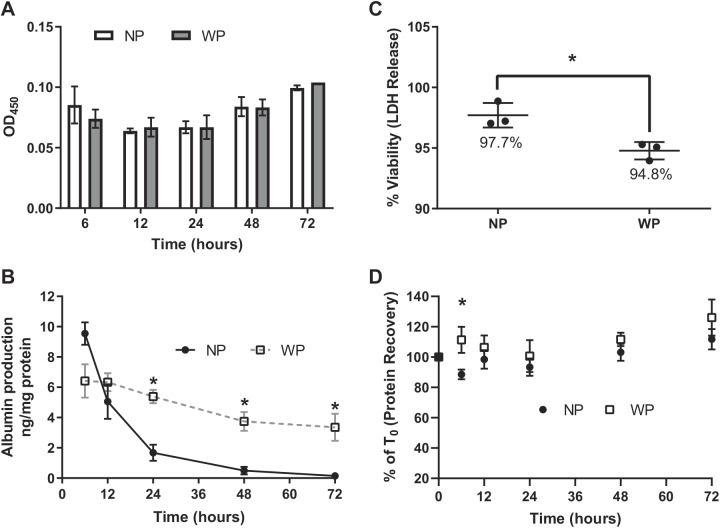
Effects of pressure on viability and functionality of hepatocytes over time. *A*: results of the water-soluble tetrazolium salt (WST-1) viability assay. OD, optical density. *B*: assay of albumin production. Prior to aspiration of medium, a sample of medium was taken and stored at −80°C, and albumin concentration was determined using a commercial sandwich ELISA kit. *C*: lactate dehydrogenase (LDH) release after 48 h of incubation with pressure (WP) or without pressure [no pressure (NP)]. *D*: percent protein recovery over time, with *T*_0_ set as 100%. Values are means (SD) of 3 separate experiments (*n* = 3). For *A, B*, and *D*, significance was determined by 2-way ANOVA and Bonferroni’s multiple-comparison test between pressure groups at each time point. For *C*, significance was determined by unpaired *t* test. *P < 0.05 between groups.

To determine if application of hydrostatic pressure was compatible with long-term hepatocyte culture, viability via WST-1 and LDH release assays, functionality via albumin production, and cell loss via protein recovery assays of hepatocytes in both WP and NP conditions were tested. A two-way ANOVA on the WST-1 data (Fig. 7*A*) revealed a significant difference with time (*P* = 0.004), but not with pressure (*P* = 0.970) and did not reveal an interaction (*P* = 0.084). Furthermore, Bonferroni’s post hoc test revealed no significant difference between the NP and WP groups at each time point. Thus the viability of hepatocytes (determined by WST-1 assay) changes with time in culture but is not due to differences in pressure. Meanwhile, a two-way ANOVA on albumin production (Fig. 7*B*) detected significant effects for time (*P* < 0.001), interaction (*P* = 0.004), and pressure (*P* = 0.010). Additionally, multiple-comparison testing found a significant difference at the 24-h time point (*P* = 0.027), whereas all other comparisons were not significant (*P* > 0.05). The LDH release assay (Fig. 7*C*) showed a small, but significant, 3% change in viability after a 48-h incubation, as determined by Welch’s unpaired *t* test (*P* = 0.018). Protein recovery data (Fig. 7*D*) were significant, with both time (*P* < 0.001) and pressure (*P* < 0.001) effects but no interaction. Bonferroni’s comparison testing of NP with WP at each time point showed a significant difference between the 6-h NP and WP groups; all other comparisons were not significant. In summary, although pressure increases albumin production, it has minimal effect on the overall viability or protein recovery of cultured hepatocytes as measured by WST-1, LDH release, and Pierce BCA protein assays.

#### Effects of pressure on expression of important absorption, distribution, metabolism, and excretion genes.

**Fig. 8.**
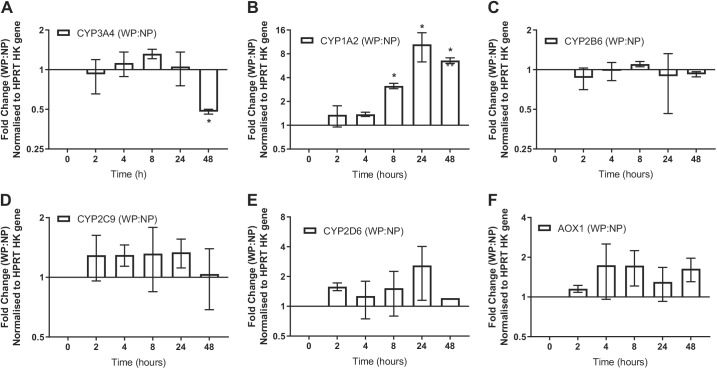
Effects of pressure on expression of 6 absorption, distribution, metabolism, and excretion (ADME) genes: cytochrome *P*-450 (CYP) 3A4 (*A*), CYP1A2 (*B*), CYP2B6 (*C*), CYP2C9 (*D*), CYP2D6 (*E*), and aldehyde oxidase 1 (AOX1; *F*). WP, with pressure; NP, without pressure [no pressure (NP)]; HPRT HK, hypoxanthine-guanine phosphoribosyltransferase 1 housekeeping gene. Values are means (SD); *n* = 3, except CYP2D6 (*n* = 2). *P < 0.05 between ΔC_T_ values (by 2-way ANOVA and Bonferroni’s post hoc test).

The effects of pressure on a selection of important absorption, distribution, metabolism, and excretion (ADME) genes were investigated. Data were normalized to the HPRT housekeeping gene to generate a ΔC_T_ value and displayed as a 2^−ΔΔCT^ value, with the NP group at each time point used as the calibrator. A two-way ANOVA and Bonferroni’s comparison testing were used to compare NP with WP at each time point using the ΔC_T_ values. In Fig. 8*A*, CYP3A4 increases ≤1.5-fold at 8 h, decreases to baseline at 24 h, and reaches a significant (*P* < 0.001) 2-fold reduction in mRNA at 48 h. CYP1A2 (Fig. 8*B*) shows a steady increase in mRNA under pressure relative to the NP control, becoming significant at 8 h (*P* = 0.001) and maintaining this significance at both 24 and 48 h in culture; the maximal change of ~10-fold occurs at 24 h. In contrast to CYP3A4 and CYP1A2, CYP2B6 (Fig. 8*C*) appears to be entirely unaffected by pressure application, in that pressure application neither increases nor decreases the number of mRNA transcripts. CYP2C9 (Fig. 8*D*), CYP2D6 (Fig. 8*E*), and AOX1 (Fig. 8*F*) generally retain a slightly higher transcript level under pressure, although the response is highly variable and not statistically significant. Overall, this points to a CYP1A2-specific increase under hydrostatic pressure, and investigation into whether this increase relates to a significant increase in activity was required.

#### Pressure improves activity of CYP1A2 in human hepatocytes and HepaRG and HepG2 cells after 48 h of preincubation with pressure.

**Fig. 9.**
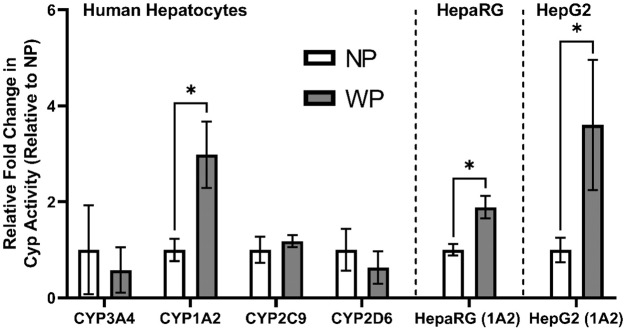
Metabolism of luminescent substrates by primary human hepatocytes (PHH) after 48 h of preincubation without pressure [no pressure (NP)] or with pressure (WP). To confirm upregulation of cytochrome *P*-450 (CYP) 1A2 (CYP1A2) activity in human hepatocytes and test potential involvement of aryl hydrocarbon receptors (AhR), CYP1A2 activity was measured in both HepaRG and HepG2 cell lines. Values are means (SD) of 2–3 repeats; *n* = 3 for CYP1A2 and *n* = 2 for all other isoforms. *P < 0.05 (by unpaired *t* test).

P450-Glo, a luminescence-based measure of metabolism, was utilized to determine if changes in mRNA translated to an increase in activity. Human hepatocytes (Fig. 9) were assessed for differences in the metabolic activity of CYP3A4, CYP1A2, CYP2C9, and CYP2D6 after 48 h of preincubation under pressure. Only CYP1A2 demonstrated a significant fold change under pressure, in agreement with qPCR results. To verify that the increase in CYP1A2 was not specific to human primary cells, we also tested HepaRG and HepG2 cells, which are considered good hepatocyte model cell lines for assessment of enzyme inductions and have conserved AhR signaling pathways (27). As such, they are often used to investigate potential drug-drug interactions (20). CYP1A2 activity was measured after 48 h of incubation under pressure in both cell types to determine if the CYP1A2 increase in human hepatocytes was retained. The similar significant fold increase in CYP1A2 response between cell types [~3-fold (*P* = 0.009) in hepatocytes, ~2-fold (*P* = 0.004) in HepaRG cells, and ~3.6-fold (*P* = 0.031) in HepG2 cells] suggests that the pressure-mediated mechanism of CYP1A2 induction is conserved between hepatic cell types. As both HepaRG and HepG2 cells retain their AhR signaling and AhR is a known regulator of CYP1A2, it was investigated for mechanosensitivity.

#### Effects of AhR agonist and antagonist on the pressure-mediated increase in CYP1A2 expression and activity.

**Fig. 10.**
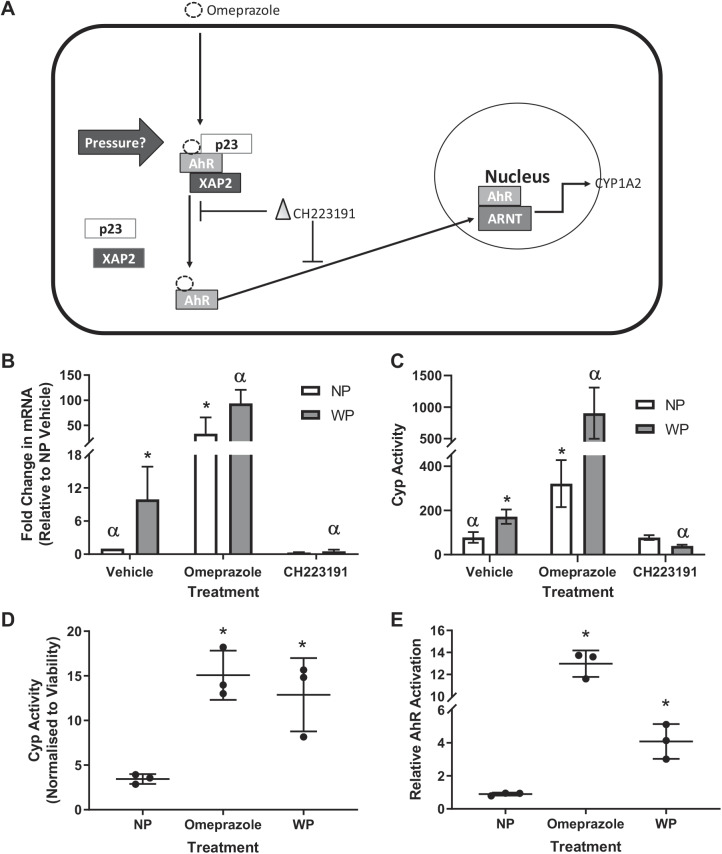
*A*: overview of experimental design. Unlike tetrachlorodibenzodioxin (TCDD), omeprazole binds to the aryl hydrocarbon receptor (AhR) with only moderate potency; it also exhibits none of the toxic side effects of TCDD (46). CH223191 is a potent antagonist that blocks AhR activation. The molecular mechanism remains unknown, but it is thought that binding of CH223191 either prevents dissociation of the inhibitory complex or covers the nuclear localization signal (NLS), blocking nuclear translocation. CH223191 at 10 µM also had no effect on cell viability in HepG2 cells (9, 66). XAP2, hepatitis B virus X-associated protein 2. *B* and *C*: relative change in expression of CYP1A2 (*B*) or CYP1A2 metabolism (*C*) in hepatocytes preincubated with pressure (WP) or without pressure [no pressure (NP)] and treated with DMSO (vehicle), omeprazole (50 µM), or CH223191 (10 µM) for 48 h. Values are means (SD) of 3 repeats (*n* = 3). *D* and *E*: activity of CYP1A2 and activation of AhR in a luciferase reporter cell line. Values are means (SD) of 3 repeats (*n* = 3). *P < 0.05 vs. NP vehicle, ^α^*P* < 0.05 vs. WP vehicle (by Bonferroni’s post hoc test). Data in *C* were log-transformed to meet variance and normality requirements of statistical tests, but linear data are displayed.

As CYP1A2 is regulated by a receptor pathway different from the other CYP isoforms examined, it was hypothesized that the pressure-induced upregulation of CYP1A2 was mediated by AhR. To test this theory, pressurized and nonpressurized hepatocytes were incubated with a known AhR inducer, omeprazole, as a positive control (33), the AhR antagonist CH223191 (8, 66), and the vehicle control DMSO. This was done to show that the AhR pathway remains active in monolayer culture hepatocytes and to determine if an AhR antagonist (CH223191) could block or inhibit the pressure-mediated increase in CYP1A2 expression and activity (Fig. 10, *A* and *B*). Expression changes were highly significant (Fig. 10*B*), with significant effects of treatment, pressure, and interaction (*P* < 0.001), as determined by a two-way ANOVA on ΔC_T_ values. Bonferroni’s multiple-comparison testing found significance as follows: a significant fold increase of 9.9 from NP vehicle to WP vehicle (*P* = 0.002), showing retention of pressure-mediated increases in CYP1A2 expression. Omeprazole results were as expected [significant increases in expression: 33.17-fold for NP inducer vs. NP vehicle (*P* < 0.001) and 10.57-fold for WP inducer vs. WP vehicle (*P* = 0.001)], which showed that hepatocytes cultured in this way retain their AhR signaling mechanics. The AhR antagonist had a marked effect on expression, decreasing WP expression by 18.1-fold compared with the vehicle control (*P* < 0.001), while only causing a nonsignificant 3.3-fold reduction in NP expression with respect to its vehicle control (*P* = 0.118).

P450-Glo data (Fig. 10*C*) showed a significant difference between pressure, interaction, and treatment (*P* < 0.001, *P* < 0.001, and *P* = 0.017, respectively). Bonferroni’s post hoc test showed a significant upregulation of WP vehicle compared with NP vehicle, with a fold change of 2.2 (*P* = 0.027). Omeprazole caused a significant upregulation from vehicle of 4.1- and 5.2-fold for NP (*P* < 0.001) and WP (*P* < 0.001), respectively. There was a −4.3-fold change from WP vehicle to WP coincubated with CH223191 (*P* < 0.001) compared with a fold change of 1.0 (i.e., no change) between NP vehicle and NP coincubated with CH223191 (*P* > 0.999).

Furthermore, to confirm the results, a luciferase-transfected AhR reporter HepG2 cell line (DRE-1A2) was also used to assess whether AhR was activated in response to pressure. DMSO vehicle and omeprazole were used as negative and positive controls, respectively.

For CYP1A2 activity (Fig. 10*D*), one-way ANOVA showed significant differences between treatment groups (*P* = 0.006), and Bonferroni’s adjusted *t* test showed significant differences for NP vs. omeprazole (*P* = 0.005) and NP vs. WP (*P* = 0.014). Lastly, for AhR activation (Fig. 10*E*), one-way ANOVA showed significant differences between treatment groups (*P* < 0.001), with Bonferroni’s adjusted *t* test revealing significant differences of ~13-fold for NP vs. omeprazole (*P* < 0.001) and ~4-fold for NP vs. WP (*P* = 0.011).

Together, these results demonstrate that the application of hydrostatic pressure leads to an increase in activation of the AhR signaling pathway, which results in a significant upregulation of CYP1A2 expression and activity. Furthermore, this increase is abolished by addition of the AhR antagonist CH223191, implicating AhR in this novel mechanosensitive signaling pathway.

## DISCUSSION

### 

#### Could vessel organization in the liver lobule impose pressure on hepatocytes?

The apparent density (Fig. 4*G*) and surface area (Fig. 4*H*) of vessels increase by ~10% and 250%, respectively, from the periphery toward the central vein. This suggests that unless the relative blood pressure within sinusoidal vessels drops to a similar extent, a radial pressure will build up, compressing the hepatocyte plates. However, it has been suggested that the drop in pressure between the periphery and the central vein is ∼15–20% (14), suggesting, in turn, that one order of magnitude (×10) is missing, indicating that a compression of the plates may occur. On these premises, the model shown in Fig. 5, which indicates a compression of hepatocytes between vessels, was suggested. Although bile contractility was reported by Oshio and Phillips (48) and, more recently, by Meyer et al. (39), the model presented in the present study could also provide a mechanical reason for bile flow in the opposite direction of blood flow. The accepted theory is that ions released into the bile duct move the bile via osmosis (4). Nevertheless, there are other possible explanations: a gradient of compression of hepatocyte plates being greater around the central vein and less toward the periphery could press against the bile duct from the center to the periphery, generating a physical stress and allowing the bile to flow in the opposite direction of blood flow.

Finally, differences between peripheral and pericentral sinusoids have been observed in rat and mouse liver. It has been reported that the size of the sinusoidal vessels increases toward the central vein (10, 32, 46, 64); these data are summarized in Table 1 and are in good agreement with our data.

**Table 1.** Summary of parameters measured in different species

Species	Parameter	PP	PV	Fold Increase PV/PP	Reference
Rat	Diameter, µm	6.4	8.3	1.30	32
	Velocity, mm/s	0.2	0.5	2.41	
Mouse	Diameter, µm	8.8	13.7	1.56	64
Rat	%Area occupied by sinusoids	26.5	38.5	1.45	10
	%Area occupied by lumen	5.5	13.0	2.36	
Rat	Total SA, µm^2^	50,767	85,155	1.68	46
	Mean SA, µm^2^	23,163	41,301	1.78	
	Total HA, µm^2^	370,431	337,165	0.91	
	Sinusoidal perimeter, µm^2^	24,909	36,410	1.46	
Pig	HA/nuclei, µm^2^/nuclei	190.1	170.5	0.90	Present data
	Vessel area/BP, µm^2^/BP	12.1	28.1	2.33	
	%Area occupied by sinusoids	10.1	19.3	1.92	
	%Area occupied by hepatocytes	89.9	80.7	0.90	

SA, sinusoidal area; HA, hepatocyte area; BP, branch point; PP, periportal; PV, perivenous.

#### Hydrostatic pressure effects on morphology, viability, and functionality.

Our method of applying hydrostatic pressure, which utilized a gas-permeable membrane, allowed the negation of hypoxia, a variable that is sometimes overlooked in other pressure application methods (36). Based on calculations published previously (50) and reports that hypoxia reduces the expression of both AhR and CYP1A2, in addition to most other CYP isoforms (22, 42, 61, 65), and with use of a gas-permeable membrane that allows O_2_ to diffuse directly to the cellular monolayer, we could rule out hypoxia as the reason for the data collected. Interestingly, pressure does not appear to affect overall cell morphology and viability, as seen in bright-field images (Fig. 6) and WST-1 assays (Fig. 7*A*). Although a small statistically significant increase (3%) in LDH production was detected between the WP and NP groups (Fig. 7*C*), it is unlikely that the increase is biologically significant. Additionally, it is not known whether pressure itself impacts LDH production or secretion.

Albumin production is a common method for determining hepatocyte functionality. Albumin production was better maintained in the WP than NP hepatocytes (Fig. 7*B*). While there is no measure of albumin production in hepatocytes under hydrostatic pressure in the literature, a number of studies that used shear stress within microfluidic devices or bioreactors demonstrated increases in albumin production with flow rate/shear stress (11, 17, 19). Overall, these results, like ours, suggest that hepatocyte functionality improves after the application of in vivo mechanical forces.

In conclusion, the seeming equivalence in phenotype and minimal change in viability, as determined by WST-1 and LDH release assays, along with the improved maintenance of albumin production in hepatocytes cultured under pressure, led us to conclude that long-term culture of hepatocytes in the pressure system, both with and without pressure, had no negative effects. The effects of hydrostatic pressure on the viability of various cell types have been assessed, and the results are mixed. In bladder smooth muscle cells, viability improved under 0.75- to 4-kPa pressure (15, 16, 25), whereas no detectable difference was observed in human retinal cells subjected to similar pressure (47). In contrast, some researchers reported reductions in cell viability under pressure, although the pressure applied was four times (4 kPa) that used in this study (30). Whether these discrepancies were due to the use of different species/cell types and/or methods is not known; however, a review of the various methods commonly used to apply hydrostatic pressure has shown differences in cell proliferation and viability due to cell type and amount loaded (38).

#### Hydrostatic pressure: CYP expression and activity.

The mRNA expression of five CYP isoforms and AOX1, which are heavily involved in drug metabolism, was assessed. CYP3A4 showed a twofold decrease in mRNA after a 48-h incubation under pressure, while earlier time points showed little difference (Fig. 8*A*). CYP1A2 showed what appeared to be an inductive response to pressure, increasing ~10-fold after 24 h (Fig. 8*B*) and maintaining this level at the 48-h point. It should be noted that CYP3A4 and CYP2D6 in culture are known to experience a rapid initial decrease until 48 h, at which point an upturn in activity and expression usually begins (35). It is also noted that the other CYP isoforms generally decline, but with no subsequent upturn (29, 35). Our results show that CYP2B6 (Fig. 8*C*) followed the expected pattern, but CYP1A2 and, to a much lesser extent, CYP2C9 (Fig. 8*D*) maintained reasonably steady rates of activity and expression before an upturn. However, we found that only CYP1A2 showed a robust and reproducible induction of mRNA expression and activity in response to hydrostatic pressure, while CYP3A4 showed a small decrease in mRNA expression. A possible explanation for the downregulation of CYP3A4 is the possibility of negative cross talk between AhR and pregnane X receptor (PXR). Indeed, it has been observed that coincubation of AhR ligands and rifampicin reduces rifampicin-mediated CYP3A4 expression in HepaRG cells and human hepatocytes and that siRNA knockdown of AhR increases basal CYP3A4 expression (52). This suggests that the upregulation of AhR, which controls CYP1A2 expression, has a negative cross-talk effect on PXR, which regulates CYP3A4 (52). This could explain the downregulation of CYP3A4.

To assess whether the change in CYP1A2 mRNA also leads to functional increases in CYP activity, P450-Glo was employed to assess changes in activity. Hepatocytes and HepaRG and HepG2 cells exhibited significant increases in activity when preincubated under pressure for 48 h (Table 2). Although there are no data in the literature on the effects of hydrostatic pressure on CYP expression and activity in hepatocytes, microfluidic applications have yielded similar results (11, 17, 19, 28, 41, 53, 60).

**Table 2.** Increase in CYP1A2 metabolism under pressure

Cell Type	Fold Change	*P* Value
PHH	3.0	0.009
HepaRG	2.0	0.004
HepG2 (WT)	4.3	0.031
DRE-1A2	3.7	0.017

CYP1A2, cytochrome *P*-450 1A2; PHH, primary human hepatocytes.

#### Hydrostatic pressure improves expression and activity of CYP1A2 via an AhR-mediated mechanism.

The isoform-specific increase in CYP1A2 expression, while the other drug-metabolizing isoforms were mostly unaffected (Fig. 9), led to the conclusion of differential regulation. As CYP1A2 is regulated differently from the other CYP isoforms, it is under the control of AhR, while others are controlled by PXR, constitutive androstane receptor, or a combination of these regulators (40). To determine if AhR was responsible for the differences, we devised a form of induction assay that involved testing the traditional in vitro inducer (omeprazole), an AhR antagonist (CH223191), and vehicle control for changes in expression and activity.

As expected from previous data, the WP vehicle group results were significantly higher for both expression (~10 fold) and activity (~2 fold). Also, as expected, omeprazole induced both CYP1A2 gene expression and activity levels and functioned as a positive control in the assay. Also, the AhR inhibitor (CH223191) virtually abolished the pressure-mediated expression and activity effect, whereas there was no significant change in NP expression or activity in the presence of the AhR inhibitor.

A HepG2 cell line stably transfected with a luciferase-linked AhR (DRE-1A2) promoter was also used to assess the activation of the receptor. The prototypical in vitro inducer omeprazole was also added as a positive control to prove that the genetically modified cell lines retained their induction capability. These results confirmed that pressure was capable of activating AhR signaling and upregulating CYP1A2 activity.

One tentative explanation for the specific effect of pressure on AhR is that the β-catenin/Wnt pathway is thought to interact with and potentially comodulate AhR (24). The mechanosensitivity of AhR could be due to its interaction with the β-catenin/Wnt pathway via the nongenomic pathway (34) and, not necessarily, direct activation. The complex interaction between AhR and β-catenin/Wnt has been alluded to on numerous occasions (5, 24, 51). Further work would be needed to clarify the exact signaling mechanisms involved. Furthermore, the involvement of AhR could have wider implications, including increasing the expression of phase II metabolic enzymes, such as UDP-glucuronosyltransferases, and transporters, such as organic anion-transporting polypeptide 1B1 (40).

#### Shear stress experiments: is shear force impacting the cells?

There is no literature relating AhR induction to hydrostatic pressure, although there is some evidence that microfluidics upregulate AhR (9, 41). It is possible, therefore, that the upregulation was due to the incidental addition of hydrostatic pressure in the shear stress experimental design or that hepatocytes sense simply a force, rather than being specifically shear stress-sensitive.

Shear stress experiments involving hepatocytes have often reported CYP1A2 expression and/or activity and albumin production among their major findings (11, 17, 19, 28, 41, 53, 60). However, often hepatocytes are enclosed in collagen sandwich configurations, which typically use collagen at 1–3 mg/mL (11, 18). Collagen at these concentrations has a shear strength of 50−550 dyn/cm^2^, meaning that it is able to completely resist the shearing force of anything <50 dyn/cm^2^ (at 1 mg/mL overlay concentration) (57).

This shear strength of collagen is often many times higher than the shear stress applied in these studies; thus it is possible that the improvements observed in these experiments are due to the penetration of drug compounds, movement of toxic compounds away from the cells, or other effects of flowing media, rather than application of shear stress to the cells. Furthermore, it has been argued that there might be inadvertent introduction of hydrostatic pressure within the system; this is easiest to visualize in pumps that use hydrostatic pressure to drive fluid motion (45, 62) but is quite possible in other setups (38). In these setups, it is possible that hydrostatic pressure is applied at magnitudes many times greater than the shearing force (commonly used shearing force is 0.6 dyn/cm^2^, which equates to just 6e^−5^ kPa), whereas a pump positioned just 10 cm above the cell could apply ~1-kPa pressure.

#### Conclusion.

Although the underprediction of hepatic metabolic clearance by hepatocyte cultures is well known, there has recently been a renewed focus on discovering the cause. With the advent of microfluidic systems, the effects of mechanical forces on hepatocyte phenotype and expression have taken center stage. The results presented here demonstrate that a mechanical force, in this case hydrostatic pressure, can activate AhR and upregulate CYP1A2, while having beneficial effects on albumin production and minimal impact (negative or otherwise) on cellular viability. These findings are important, because they describe a new and novel link between hydrostatic pressure and the control of CYP metabolism and reinforce the limitations of standard two-dimensional cultures for drug clearance prediction and highlight a key improvement opportunity by introducing aspects of hepatic physiology.

## GRANTS

This work was funded by the Biotechnology and Biological Sciences Research Council and Vertex Pharmaceuticals Europe Ltd. (ID 501100000268).

## DISCLOSURES

No conflicts of interest, financial or otherwise, are declared by the authors.

## AUTHOR CONTRIBUTIONS

L.B., P.S., S.W.P., and C.R. conceived and designed research; L.B. performed experiments; L.B., S.W.P., L.A., and C.R. analyzed data; L.B., S.W.P., H.R.M., L.A., P.L., and C.R. interpreted results of experiments; L.B. prepared figures; L.B. and C.R. drafted manuscript; L.B., S.W.P., H.R.M., L.A., P.L., and C.R. edited and revised manuscript; L.B., S.W.P., H.R.M., L.A., P.L., and C.R. approved final version of manuscript.
